# Fast discovery and visualization of conserved regions in DNA sequences using quasi-alignment

**DOI:** 10.1186/1471-2105-14-S11-S2

**Published:** 2013-09-13

**Authors:** Anurag Nagar, Michael Hahsler

**Affiliations:** 1 Department of Computer Science and Engineering, Southern Methodist University, Dallas, TX 750205, USA; 2 Department of Engineering Management, Information, and Systems, Southern Methodist University, Dallas, TX 750205, USA

## Abstract

**Background:**

Next Generation Sequencing techniques are producing enormous amounts of biological sequence data and analysis becomes a major computational problem. Currently, most analysis, especially the identification of conserved regions, relies heavily on Multiple Sequence Alignment and its various heuristics such as progressive alignment, whose run time grows with the square of the number and the length of the aligned sequences and requires significant computational resources. In this work, we present a method to efficiently discover regions of high similarity across multiple sequences without performing expensive sequence alignment. The method is based on approximating edit distance between segments of sequences using *p*-mer frequency counts. Then, efficient high-throughput data stream clustering is used to group highly similar segments into so called quasi-alignments. Quasi-alignments have numerous applications such as identifying species and their taxonomic class from sequences, comparing sequences for similarities, and, as in this paper, discovering conserved regions across related sequences.

**Results:**

In this paper, we show that quasi-alignments can be used to discover highly similar segments across multiple sequences from related or different genomes efficiently and accurately. Experiments on a large number of unaligned 16S rRNA sequences obtained from the Greengenes database show that the method is able to identify conserved regions which agree with known hypervariable regions in 16S rRNA. Furthermore, the experiments show that the proposed method scales well for large data sets with a run time that grows only linearly with the number and length of sequences, whereas for existing multiple sequence alignment heuristics the run time grows super-linearly.

**Conclusion:**

Quasi-alignment-based algorithms can detect highly similar regions and conserved areas across multiple sequences. Since the run time is linear and the sequences are converted into a compact clustering model, we are able to identify conserved regions fast or even interactively using a standard PC. Our method has many potential applications such as finding characteristic signature sequences for families of organisms and studying conserved and variable regions in, for example, 16S rRNA.

## Background

With the development of Next Generation Sequencing techniques, there has been a massive increase in the number of sequences available. Analyzing such a large volume of sequence data presents a major computational challenge, especially since it often involves finding an optimal alignment between the sequences as a first step. Multiple Sequence Analysis (MSA), which is most commonly used for aligning a set of sequences, is computationally very expensive. In many bioinformatics applications (e.g., BLAST [1], BAlibase [2], T-Coffee [3], MAFFT [4], MUSCLE [5,6], Kalign [7] and ClustalW2 and ClustalX2 [8]), sequence alignment and MSA play a critical role. Finding the optimal alignment for a large set of sequences that may be related by function, evolution, or structure is a computationally complex task and often involves use of high performance computing servers and resources.

Alternative approaches involve creating statistical signatures from nucleotide composition frequencies. These so-called alignment-free methods [9] are more efficient both in terms of processing time and storage requirements as they work with compact signatures and not the entire set of sequences. These methods also scale well for whole genome phylogenetic analysis [10] which is an improvement over existing methods. However, these methods do not retain any of the important local information, such as the GC content in particular areas of the sequences. Several other methods have tried to characterize sequences based on the repeated presence of certain shorter patterns [11,12]. These methods are mostly used as heuristics for identification of smaller set of sequences.

It is well known that different regions of DNA sequences have different roles. For example, some regions are responsible for protein coding and are known as coding regions [13] while others are conserved across related species and can be an indication of evolutionary similarity. Thus, a flexible approach to sequence analysis is needed that can take advantage of computational efficiency of alignment-free techniques while still taking into account the unique properties of different regions of sequences.

In this work, we propose a novel method for discovering conserved regions across multiple sequences. Our method is based on position-sensitive word frequency analysis and uses high efficiency data stream clustering to find regions with similar word frequency distributions across multiple sequences. We refer to clusters as *quasi-alignments*, since a similar word frequency distribution in a cluster also means that the underlying segments are likely to be very similar. For the found clusters, we also retain important metadata such as the position of the clustered segments in the original sequences and the GC-content. This approach has previously been used for phylogenetic classification [14]. This paper expands our preliminary investigation into discovering similar segments [15] by developing a more rigorous theory of quasi-alignment, improved visualization and an expansion to the species level. We show how quasi-alignment can be used to quickly and efficiently discover conserved regions across multiple sequences. Finding stretches of identical sequences at the species level is useful for various applications including sequence identification and DNA barcoding [16].

## Quasi-alignment via position-sensitive ***p***-mer clustering

### Constructing position-sensitive frequency vectors

In this section, we present the foundation for the analysis and clustering framework. The basic unit of analysis for our case is a word inside a segment of a sequence. In the case of global frequency analysis used by alignment-free methods [9], a word of length *p *is referred to as an oligomer or *p*-mer. In case of DNA sequences, the words are formed from the set of alphabet {*A, C, T, G*}. In our analysis, we are interested in the distribution of words within specific regions (segments) of a sequence.

**Definition 1**. *Given a DNA sequence × of length L, a **segment **S_i,l _is defined as a subsequence starting at position i and having length l, where l < L − (i − *1*)*.

Next, we define the distribution of words within a segment.

**Definition 2**. *Given a segment S_i,l_, we define NSV_i,l _= *〈*f*_1_, *f*_2_, . . . , *$f_{4^{p}}$*〉 *as a vector of length *4*^p ^where each element f_i_, i = *{1, 2, . . . , 4*^p^*}*, represents the count of a possible p-mer in the segment. This vector represents the word frequency distribution in S_i,l _and is referred to the segment's **Numerical Summarization Vectors (NSV)***.

**Figure 1:**
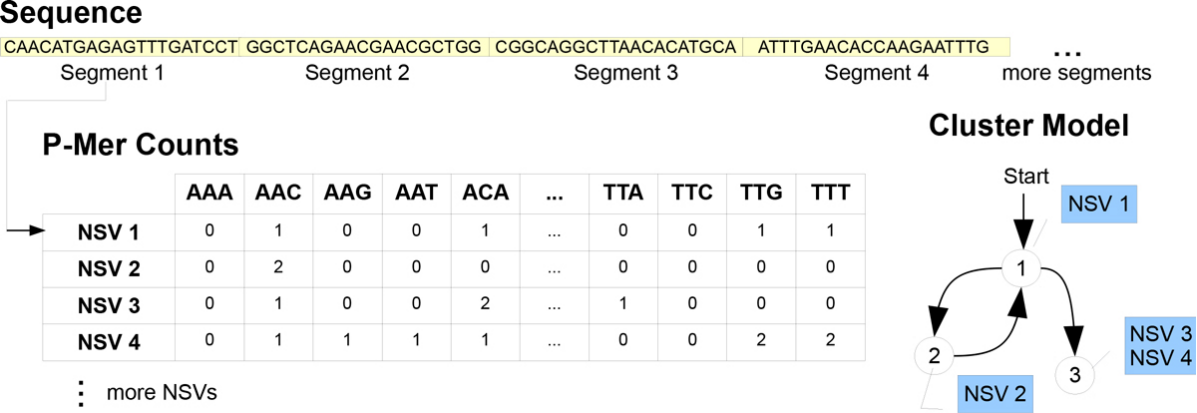
**Process of creating a GenModel**. GenModel is created by dividing the sequence into equal sized segments and evaluating word frequency distributions, called Numerical Summarization Vectors (NSVs), for each segment. This example shows word size of 3. The model is created by comparing each new segment's frequency profile with existing clusters. The segment is assigned to the closest cluster that is within a threshold distance, if no such cluster is available, a new cluster is created with the segment as the first member. Here NSV3 and NSV4 are close enough to be assigned to the same cluster.

The set of NSVs for an entire sequence can be created by partitioning it into equal sized segments that may or may not overlap. For example, a sequence of length 1500 base pairs (bp) can be divided into 15 segments each of length 100 without any overlap between them. The word frequency distribution will later be used to find similar segments. Thus, the word size parameter *p *controls how well the similarity between segments is approximated with larger values leading to better approximation while smaller values lead to faster computation. We find that *p *= 3, i.e. we count the occurrence of tri-mers within a segment, produces good results while creating NSVs of length 4^3 ^or 64. Cutting a sequence into segments and then creating NSVs is illustrated in Figure 1. We currently do not take into account any unknown characters, such as "N" that may be present due to sequencing errors or ambiguous sequencing.

### Data stream clustering

After constructing NSVs for the entire set of sequences to be examined, we use high-throughput data stream techniques [17] to cluster similar segments. We consider each NSV as a data point in a stream of consecutive NSVs. The clustering algorithm then adds one NSV after the other to the cluster model by adding it to an existing cluster if it is within a user defined clustering threshold from its center or otherwise creating a new cluster with the NSV as its first member. This idea is illustrated in Figure 1. In addition to the clusters we also retain order information in the form of a directed graph (shown in Figure 1). The exact clustering procedure is discussed in [18].

**Definition 3**. *A **GenModel **M is defined as a directed graph *$G=\langle C,E\rangle$*where the vertices are the set of clusters *$C=\left\{C_{1},C_{2},...,C_{m}\right\}$*of NSVs and the edges E represent the ordering of the NSVs in the sequences. Each cluster contains metadata such as location in the original sequence and the sequence IDs*.

We can now reframe the problem of alignment as the problem of finding similar subsequences (segments) via clustering.

**Definition 4**. *Each cluster C in a GenModel M represents a set of similar segments and is referred to as a **quasi-alignment **and the segments are said to be **quasi-aligned***.

By using clustering, we avoid the expensive alignment process and yet obtain information about local similarity between multiple sequences. The data stream clustering algorithm makes just one single pass through the segments and thus it has a linear time complexity in terms of the length and number of sequences. Also, new sequences can be added very efficiently to an existing model.

### Similarity measures for clustering

Clustering algorithms use similarity measures for comparing individual data points. In our case, the sequence data is converted into fixed dimension NSVs representing the word frequency distribution within segments. NSVs can be compared using standard measures for vectors such as Manhattan distance, Euclidean distance, squared Euclidean distance, Kullback-Leibler discrepancy and Mahalanobis distance [9]. They can also be compared using other measures such as the number of shared words between two segments. For example, Simrank [19] compares the number of matching *p*-mers (typically with *p *= 7) for fast sequence search.

The distance between NSVs can be related back to the difference between the original sequence strings also. The difference between two sequences is measured in terms of edit distance [20], which is the minimum number of point mutations required to change one sequence into another. A point mutation can be an insertion, deletion, or substitution. Ukkonen [21] has proposed that the edit distance between two strings can be approximated by the Manhattan distance between their *q*-gram profiles (which in our case will be the *p*-mer profile). The Manhattan distance between two frequency vectors *x *and *y *obtained from the segments *s_x _*and *s_y _*is defined as:

(1) $$d_{\text{Manhattan}}\left(x,y\right)=\overset{4^{p}}{\underset{i=1}{\sum }}\;\left|x_{i}-y_{i}\right|$$

The Manhattan distance simply computes the number of *p*-mers that are different between the two sequences. It can be shown that the Manhattan distance gives a lower bound for the edit distance between the two segments.

(2) $$d_{\text{Edit}}\left(s_{x},s_{y}\right)\geq \frac{d_{\text{Manhattan}}\left(x,y\right)}{2p}$$

The reasoning behind the bound above is that any insertion, deletion, or substitution in the segment will create at most *p *new *p*-mers and destroy *p *existing *p*-mers. Note that in theory it is possible to create two completely different sequences with the same *q*-gram profile (see [21]), however, this is very unlikely if we deal with biological sequences which are expected to have a certain degree of similarity (e.g., caused by conserved regions or homology).

The relationship in equation 2 can be used to determine a reasonable clustering threshold for the data stream clustering algorithm in [18] for a given word size *p*. For example, we often use a segment size of 100 bases with 3-mers and a Manhattan clustering threshold of 30. Equation 2 shows that this threshold means that the edit distance between two segments needs to be at least 30/6 = 5 to put them into two separate clusters. Note also that position specific *p*-mer frequency clustering is not restricted to using Manhattan distance, it can be used with any proximity measure defined on the frequency counts in NSVs.

### Discovery of conserved regions from GenModels

GenModels provide vital information about the similarity between segments in the form of quasi-alignments. This allows us to identify regions that are highly similar across multiple sequences. For sequences related by evolution, such as those from the same taxonomic unit, these segments are known as conserved regions.They are likely to be responsible for a particular function or provide a needed structural characteristic.

**Figure 2:**
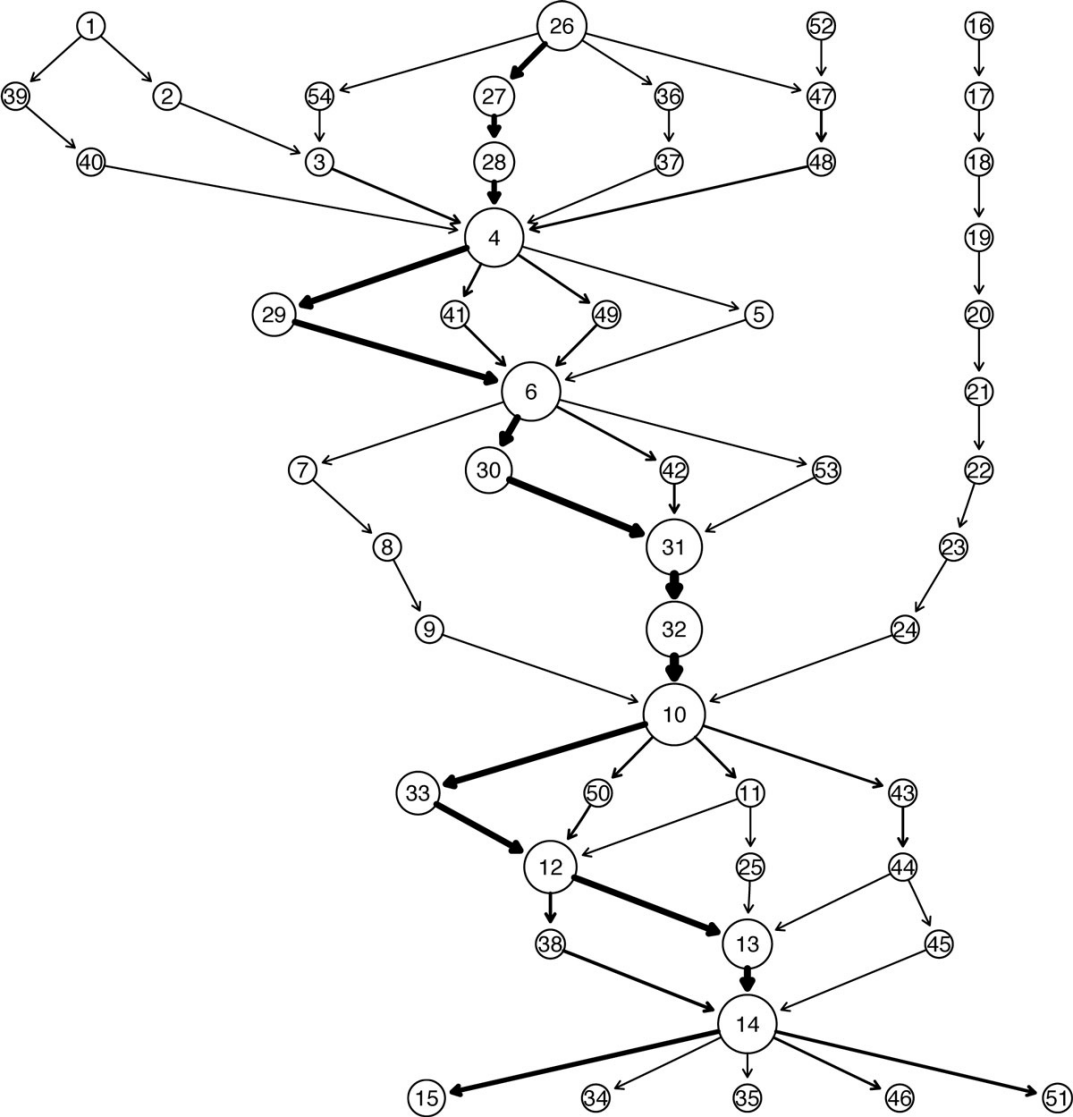
**GenModel for the phylum GN06**. GenModel of 13 16S rRNA sequences from the phylum GN06 with the circles denoting quasi-alignments (clusters of segments) with a unique id. Arrows show the preserved order information of segments in the original sequences. For example, all sequences with a segment participating in quasi-alignment 31 have the next segment in quasi-alignment 32.

As an illustration, Figure 2 shows a GenModel created from the 16S rRNA sequences belonging to the phylum *GN06*. We used the unaligned version of the sequences from the Greengenes database [22]. The available 13 sequences range in length between 1374 and 1525 bases. For building the model, these sequences were broken down into non-overlapping segments of size *l *= 100 bases each, which were then aggregated with 3-mers (*p *= 3) and clustered using Manhattan distance and a clustering threshold of 30. The resulting GenModel contains 54 clusters or quasi-alignments. The plot shows each of the quasi-alignment as circles uniquely identified by an id. The circle size is proportional to the size of the clusters (i.e., number of segments participating in the quasi-alignment) and arrows represent the direction of the transitions between them. A stronger arrow indicates that the transition occurs with a higher probability. For example, Figure 2 shows that one of the common transition paths is the quasi-alignment sequence 26 → 27 → 28 → 4 → . . . → 12 → 13 → 14 indicating that a large fraction of the sequences share some common sequence segments. In addition the plot shows that almost all sequences go through a few quasi-alignments (e.g., 4, 6, 10 and 14) which represent candidates for regions that may be highly conserved in the set of analyzed sequences. The sequences share a common higher level taxonomy (phylum) and thus we expect relatively stronger quasi-alignments as compared to random sequences. Interesting in Figure 2 is the almost completely separate path of smaller clusters starting with quasi-alignment number 16. This indicates that a single or a few sequences are significantly different from the majority of the sequences in the set. This might be due to several reasons, such as mis-classification of the sequence, or a sequencing error whereby some of the initial bases may have been removed.

**Figure 3:**
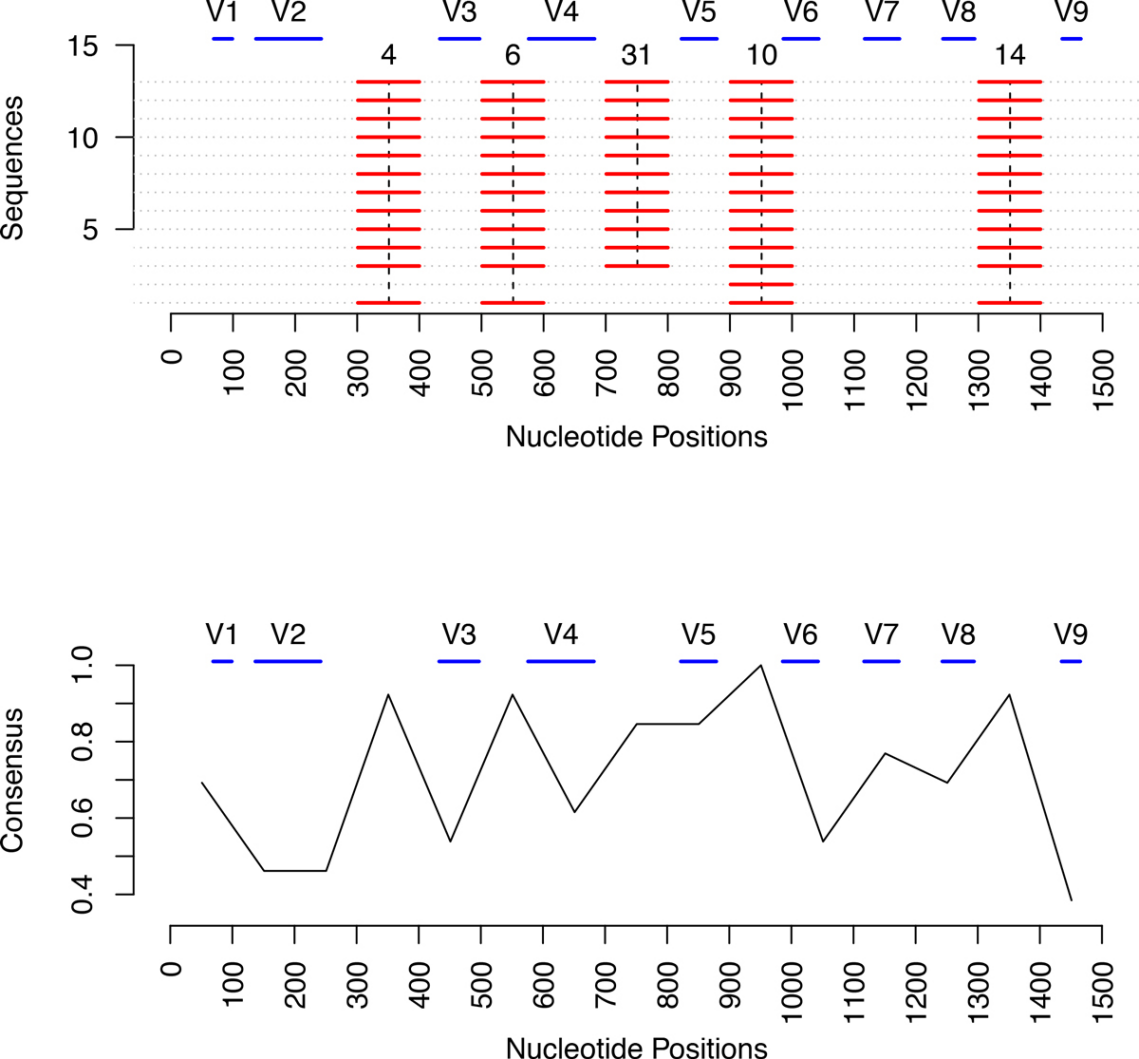
**Plot of the top 5 quasi-alignments from the phylum GN06**. The top plot shows the positions of the segments that belong to the five strongest quasi-alignments. These segments indicate well preserved regions in the sequences. The bottom plot shows the consensus among the quasi-alignments for the segment, i.e., fraction of sequences participating in the most common quasi-alignment for each segment.

In Figure 3, we visualize the largest quasi-alignments found in the GenModel along the approximately 1500 bases (x-axis). The top part of Figure 3 shows the segments grouped into the 5 most popular quasi-alignments as red horizontal lines. In this model, all red horizontal lines are exactly 100 bases long because a segment length of 100 was used. The segments that are part of the same quasi-alignment are joined by vertical dotted lines and the cluster id from Figure 2 is shown on top. We see that the well preserved segments are found in quasi-alignment 4, 6, 31, 10 and 14 which correspond to the largest clusters in the model where almost all sequences converge in Figure 2. The lower part of Figure 3 shows a measure of consensus for each segment i.e. proportion of sequences clustered into the most popular quasi-alignment. For example, it shows that all of the sequences in the nucleotide region 900-1000 converge in quasi-alignment 10. Similarly, all except one sequence converge in quasi-alignment 4, 6 and 14 for the nucleotide regions 300-400, 500-600 and 1300-1400, respectively. This is an indication that these segments are highly similar and could be conserved regions of the sequences.

**Figure 4:**
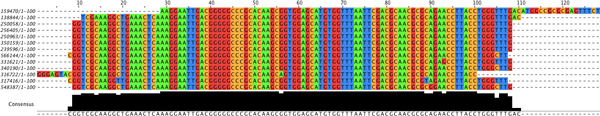
**Multiple sequence alignment of segments that are part of quasi-alignment 10 in GN06**. Multiple sequence alignment of the segments forming quasi-alignment 10 (at positions 901-1000) in the GenModel for GN06 (visualized with JalView [23]).

To validate our claim that the segments that are clustered together into a quasi-alignment are indeed highly similar, we performed traditional Multiple Sequence Alignment (MSA) on the segments that are part of quasi-alignment 10. We used Clustal [8] available through the software JalView [23,24] to perform MSA and visualize the alignment in Figure 4. The results show that the segments have an average pairwise alignment score of 0.94 (out of a maximum possible of 1.00) with a large majority of segments being almost identical and having 100% pairwise alignment. Figure 4 and the MSA results indicate that the segments in quasi-alignment 10 are indeed very similar. We have performed a similar analysis on the other quasi-alignment shown in Figure 3 and verified that the segments have a high degree of base-wise identity.

It is interesting to note that in Figure 4 some sequences have bases that are "shifted" by a certain amount. For example, the first sequence shown with id *159470*, has its bases shifted to the right by 29 bases. This can be the result of insertions/deletions (indels) in the sequences due to evolutionary processes. If this offset becomes too large, then it can interfere with clustering segments. This problem can be removed by using overlapping segments, i.e., considering many or all possible offsets. This increases the time complexity in the worst case by a fixed factor of *l *(segment length). It also makes makes visualizing quasi-alignments more complicated and therefore we will restrict the discussion in this paper to non-overlapping segment.

Further validation of the location of the found conserved regions can be obtained by looking at biological evidence available in the literature. Studies have reported that 16S rRNA contains regions that are highly conserved within each species, but variable between species. These regions are known as hypervariable regions [25,26], which are characteristic for each species, and have applications such as PCR amplification using universal primers [25]. It has also been reported that hypervariable regions are flanked on both sides by regions that are highly conserved across multiple species [27,28]. These flanking regions are conserved even for sequences exhibiting wide genomic diversity such as environmental or biological samples.

Nine identified hypervariable regions in 16S rRNA consist of nucleotides number 69-99, 137-242, 433-497, 576-682, 822-879, 986-1043, 1117-1173, 1243-1294 and 1435-1465, and are denoted by V1 through V9, respectively [25]. The sequence data of the phylum *GN06 *contains 13 sequences potentially from multiple species, which in the data have not been identified and hence are coded as *unknown *in the Greengenes database. Since the data contains several species, we would expect greater variations in the hypervariable regions than in the flanking, preserved regions. The top part of Figure 3 shows the 5 largest quasi-alignments (clusters) found for the GenModel for the sequences from *GN06*. The positions of the hypervariable regions are shown as blue lines labeled V1 through V9 at the top of the plot. It is very clear that our algorithm identifies regions that flank the hypervariable regions. For example, quasi-alignment 10 covers the nucleotide bases between the hypervariable regions V5 and V6. Similarly, quasi-alignment 4, 6, 31, and 14 cover the bases between hypervariable regions V2, V3, V4, V5, V6, V8 and V9. The plot in the lower part of Figure 3 also confirms this finding. The peaks of the plots indicate those segments that have a high consensus i.e. a strong quasi-alignment. The region between bases 900 and 1000 share a perfect consensus, i.e., all the segments belong to the same quasi-alignment. As discussed earlier, this area lies between hypervariable regions and is thus is expected to be more conserved for a sample containing multiple species.

### Implementation details

We have implemented an open source software package using the **R **framework called **QuasiAlign **which can be downloaded from http://r-forge.r-project.org/projects/mmsa/. This package has methods to quickly load a large set of sequence files, that can be in FASTA format with Greengenes [22] annotations, into a relational database and can be used to easily filter sequences belonging to any taxonomic rank. This package is built on top of a number of other packages including **Biostrings **[29] for handling sequences, and the data stream clustering package **rEMM **[30,31]. It provides a complete interface for managing sequences, creating word frequencies distributions (NSVs) and creating and analyzing GenModels. Several other useful functions, such as those for metagenomic classification, are also available. More details can be obtained from the package documentation [32].

For the analysis in this paper, we have used the default parameters for creating NSVs and GenModels. The parameters for NSVs are a segment length of *l *= 100 bases with no overlap between and segments and a word size of *p *= 3. For creating GenModels, the default is Manhattan distance with a clustering threshold distance of 30 which requires a minimum edit distance of 5 between two segments to place the into separate clusters (see equation 2 above).

### Large scale experiments

Position sensitive *p*-mer clustering can work with a set of DNA/RNA sequences or even fragments of sequences from any source. This is a valuable asset for metagenomic analysis and also fits in nicely with the requirements of Next Generation Sequencing methods. All experiments in this paper can be reproduced using the **QuasiAlign **[32] package.

### Dataset used

The method presented here for discovering conserved regions is general enough to be applied to any set of sequences. For this analysis, we used the more than 400,000 16S rRNA sequences obtained from the Greengenes project [22]. The 16S gene is widely used for phylogenetic analysis as it is highly conserved for different species of bacteria and archaea. The sequences of this gene have remained more or less constant over time and evolutionary cycles. Further, it contains several distinct regions, known as hypervariable regions, that are very specific and unique for each individual species [25] and are widespread used for sequence identification and classification. The package **QuasiAlign **allows us to directly import FASTA sequences with Greengenes annotations into a relational database for further analysis.

## Results

We processed the entire Greengenes 16S rRNA database using the default settings for creating NSVs and GenModels and then analyzed the models for interesting patterns and clusters to search for highly similar or conserved regions across multiple sequences that may be related by taxonomy.

**Figure 5:**
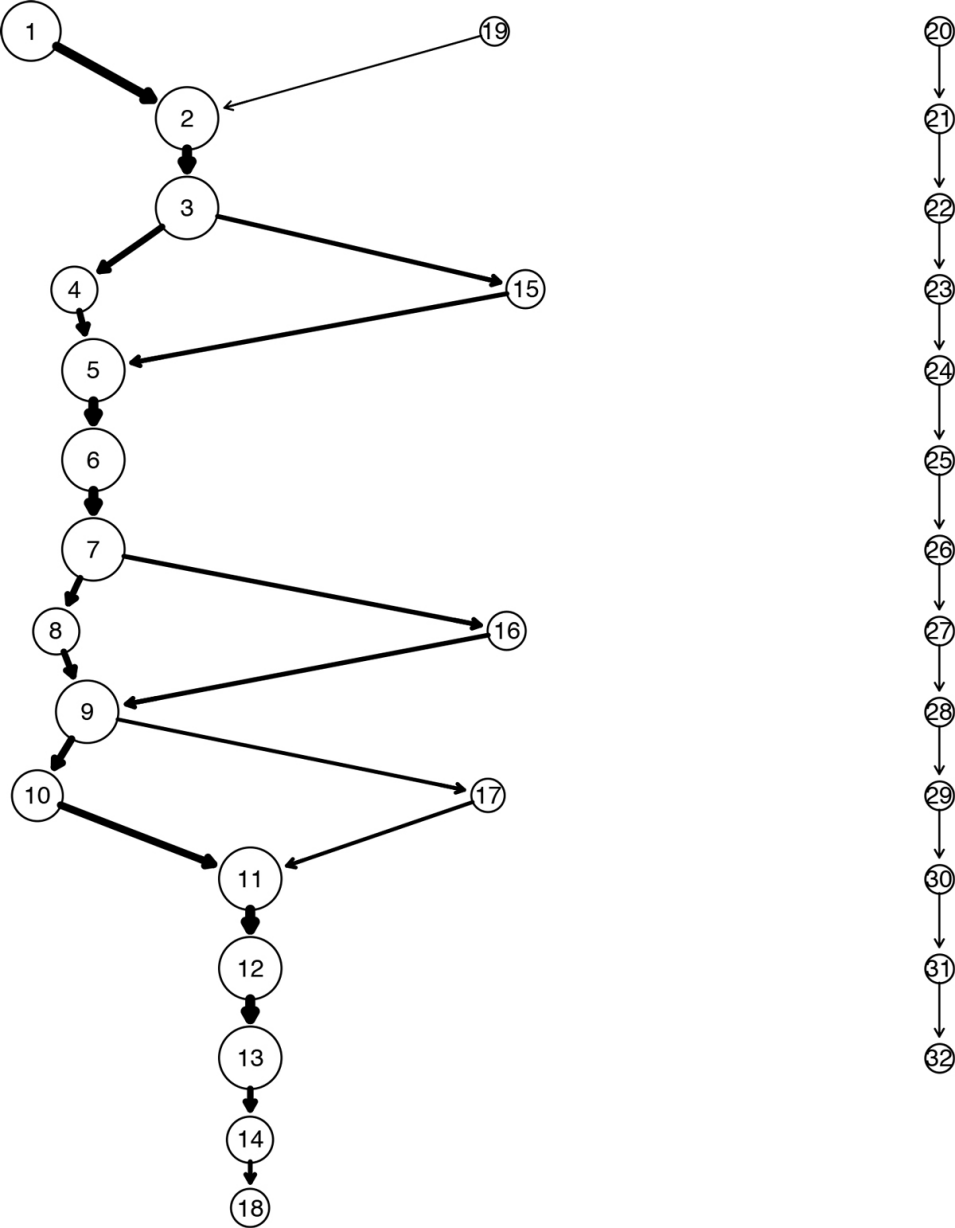
**GenModel of 16S rRNA sequences from the species Leptotrichia buccalis**.

**Figure 6:**
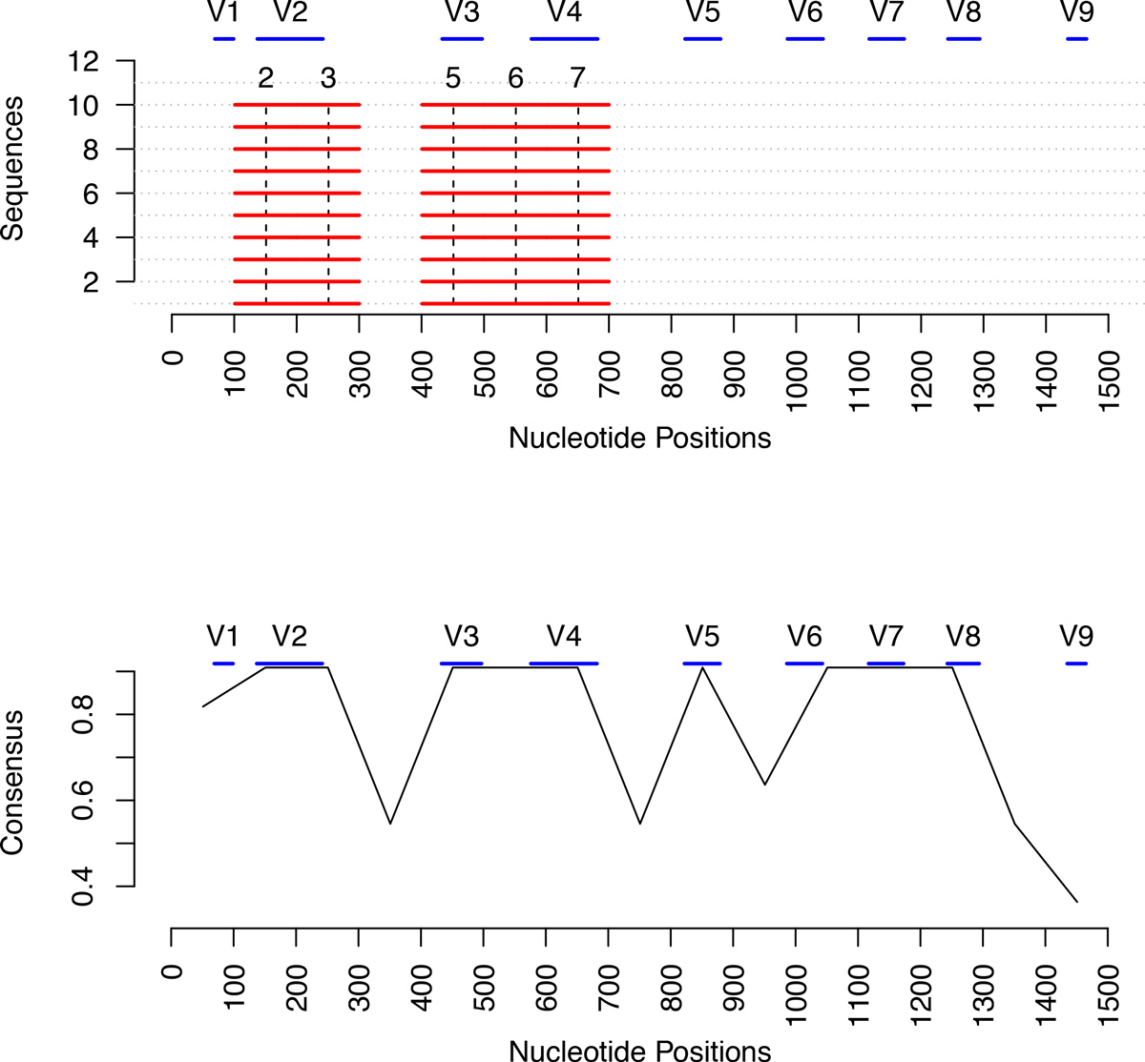
**Plot of the top 5 quasi-alignments from the species Leptotrichia buccalis**. The top plot shows the 5 strongest quasi-alignments from the species Leptotrichia buccalis. The bottom plot shows the consensus of the quasi-alignments.

**Figure 7:**
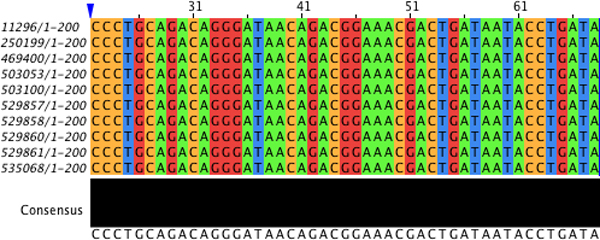
**MSA of a section of quasi-alignment 2 showing perfectly aligned bases**. Plot of the Multiple Sequence Alignment of a section of quasi-alignment 2 between bases 122 and 168 for the species Leptotrichia buccalis showing perfect alignment.

As an example, we present an analysis of the species *Leptotrichia buccalis *that belongs to the phylum *Fusobacteria *and genus *Leptotrichia*. The database contains 11 sequences of this species having lengths between 1310 and 1510 bases. We ran the position sensitive *p*-mer clustering algorithm on these sequences. The plot of the GenModel is shown in Figure 5 and the plot of the segments belonging to the 5 largest quasi-alignment is shown in Figure 6. It is easy to see that most sequences follow a similar path with the exception of one sequence that starts a totally different path starting from quasi-alignment 20 and ending at quasi-alignment 32. This outlier sequence may have its bases shifted by a certain amount due to insertions or deletions giving it somewhat of a different frequency profile. Since the sequences belong to the same species, the hypervariable regions are expected to be highly conserved. The top 5 quasi-alignments contain segments that belong to hypervariable regions V2, V3, and V4. The bottom part of Figure 6 shows that the consensus of quasi-alignments peaks at the hypervariable regions. To check these results, we also performed MSA using Clustal [8] on the segments belonging to quasi-alignments 2 and 3. The average pairwise alignment score is 0.93, and many sequences being perfectly aligned with a pairwise alignment score of 1.00. The results are shown graphically in Figure 7 for a section of the 200 bases that are perfectly aligned.

**Figure 8:**
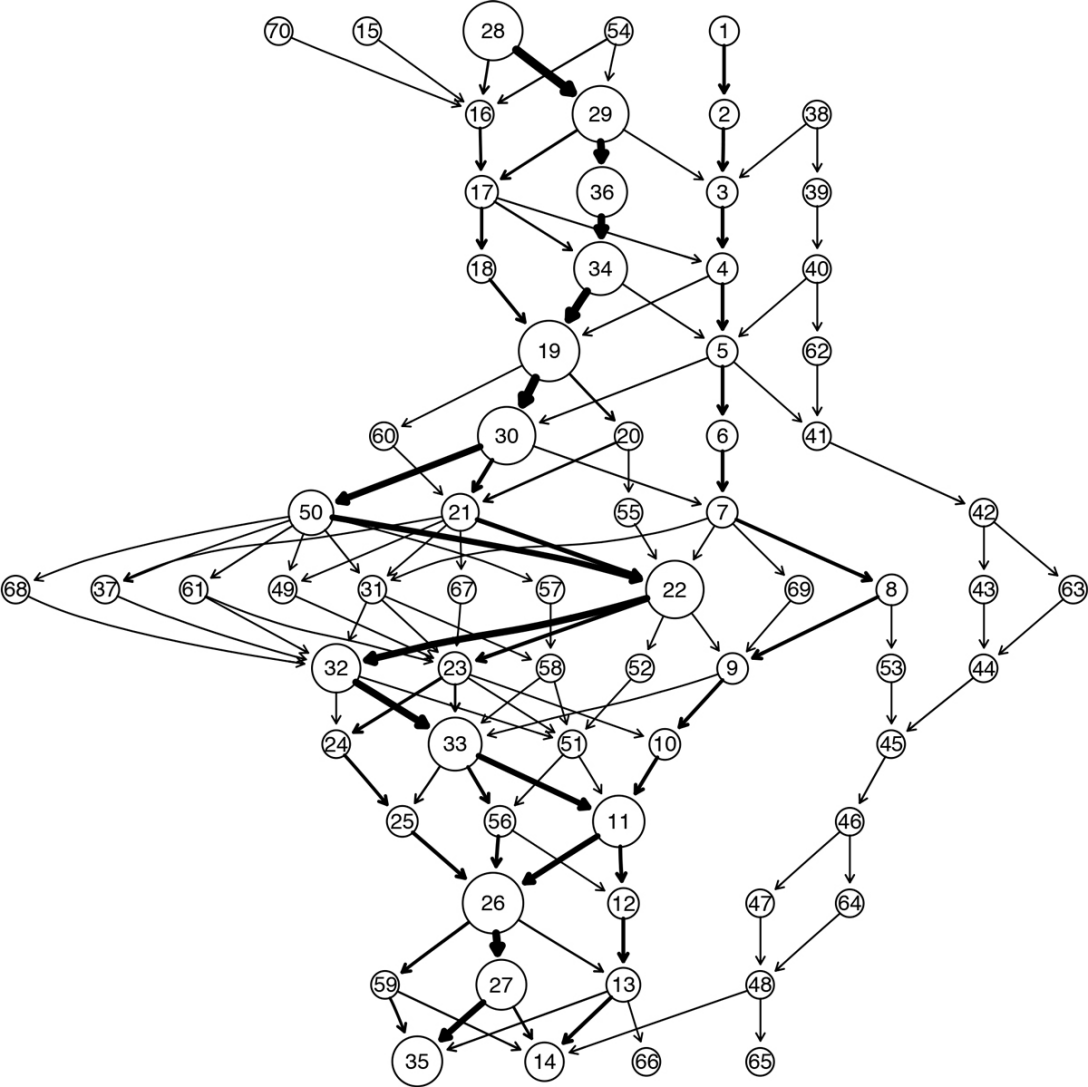
**GenModel of 16S rRNA sequences from the species Cetobacterium somerae**.

**Figure 9:**
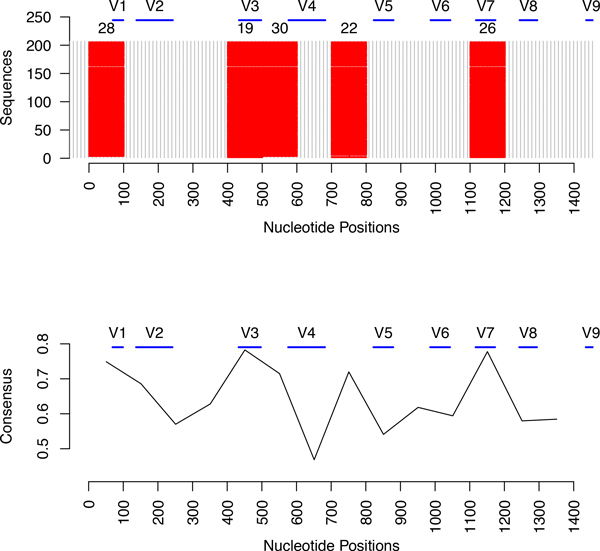
**Plot of the top 5 quasi-alignments from the species Cetobacterium somerae**. The top plot shows the 5 strongest quasi-alignments from the species Cetobacterium somerae. The bottom plot shows the consensus of the quasi-alignments.

A second example comes for the species *Cetobacterium somerae *that belongs to the phylum *Fusobacteria *and *genus Cetobacterium*. There are 207 sequences in the dataset for this species having lengths between 1335 and 1472 bases. We created the GenModel shown in Figure 8. Since there are about 20 times more sequences than for the earlier, the model is more complex and has many more clusters and related transitions. Still, we can see that there are certain clusters and transitions that are more pronounced and the most common path is 28 → 29 → 36 → . . . 26 → 27 → 35. The plot of the segments belonging to the top 5 quasi-alignments are shown in Figure 9 at the top and the consensus for the quasi-alignments is shown at the bottom. It is easy to see that the consensus mostly peaks at the location of the hypervariable regions, implying that these segments are similar and cluster together in the same quasi-alignment. We have also performed MSA on the segments belonging to the largest clusters and they have very high nucleotide similarity with several sub-regions having perfect matches.

### Applications

There are several possible applications of our method. The first is to discover regions of very high sequence alignment by limiting the search space to regions of strong quasi-alignment. For the case of the species *Leptotrichia buccalis*, we have identified in the sequences that the region between nucleotides 100 and 300 has a highly similar word frequency distribution. While this does not necessarily mean that all bases in this region will be perfectly aligned, it does indicate that this region is a good candidate for alignment. Therefore, the search space for the best alignment can be reduced from the entire sequence length to just the strongly quasi-aligned segments. This can result in substantial savings in computational resources and time and produce results more efficiently.

Another application is in the area of DNA barcoding [16], which seeks to identify species based on sequence segments that are standardized and well-conserved across sequences belonging to the same species. By using our methods, we can limit the search for DNA barcodes to those regions that are strongly quasi-aligned. For example, in case of the species *Leptotrichia buccalis*, we discovered the region between nucleotide base positions 100-300 contain highly similar sequences. Further analysis of MSA results reveals that in this region the nucleotide positions 122-168 and 183-235 are exactly identical. These regions can form the basis of a more thorough DNA barcoding analysis.

### Run time analysis

The existing methods for analyzing a group of sequences for similarity rely mostly on Multiple Sequence Alignment (MSA). Finding the optimal MSA is known to be NP hard and thus computationally challenging [33,34]. Various heuristics are currently used for MSA based analysis. Progressive alignment is a heuristic method that first constructs a guide tree based on relationships between the sequences and then builds the MSA by iteratively adding sequences from the guide tree to the alignment. The time complexity of progressive alignment is *O*(*N*^2^*L*^2^) where *N *the number of sequences having average length *L *[35].

In contrast to the above methods, position sensitive *p*-mer clustering makes just one pass through each of the sequences to create the NSVs and construct the GenModels. Thus, the time complexity of our method is *O*(*LN*) for *N *sequences of average length *L*. In addition, adding new sequences to an existing model is very easy since the use data stream clustering allows us to add new NSVs at any time.

The above advantages allow us to analyze a large set of sequences for similarity and allow easy discovery of conserved regions. Our algorithm can easily analyze the entire data set from the Greengenes [22] using a simple personal computer. Performing such an analysis using traditional MSA would require extensive server resources and computing time.

**Figure 10:**
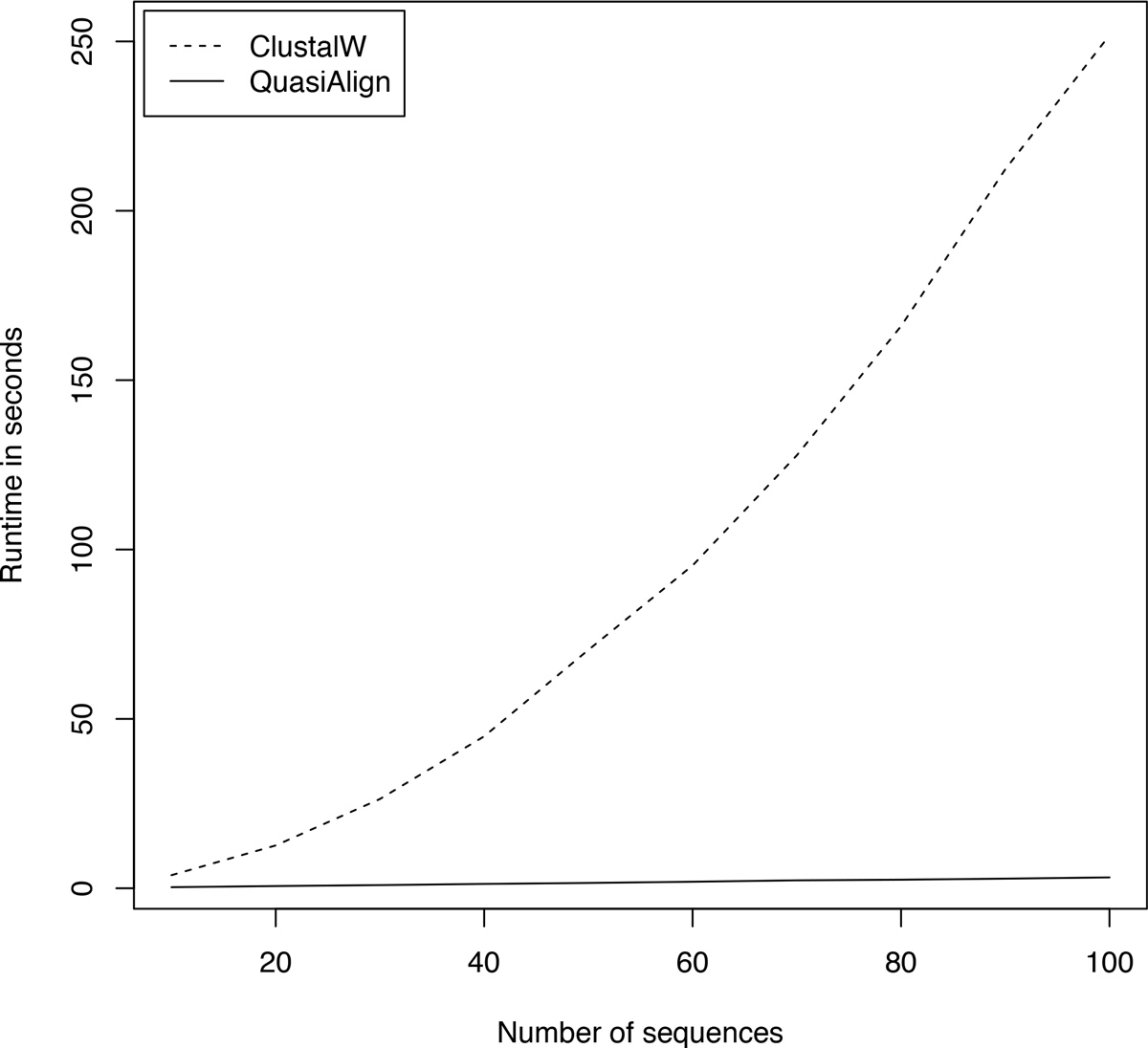
**Comparing run times of quasi-alignment and Clustal implementation of MSA**.

To compare run times of our method against MSA, we incrementally sampled between 10 and 100 sequences from the phylum *Fusobacteria *and ran quasi-alignment and the Clustal [8] implementation of MSA on them and noted the run times. The plot is shown in Figure 10. It is clear that the run time for quasi-alignment increases linearly with the number of sequences while for Clustal it grows polynomially. Because of this, quasi-alignment scales well for larger number of sequences and can provide accurate results quickly and efficiently.

## Conclusion

In this work, we have presented the foundation for quasi-alignment based on position sensitive *p*-mer clustering, a technique which applies high-throughput data stream clustering to produce GenModels, where strong clusters represent potential areas of high sequence similarity. In contrast to MSA heuristics, the runtime of quasi-alignment scales linearly in the number of sequences and the average sequence length. This allows us to process larger number of sequences efficiently. We carried out experiments for sequences consisting of single and multiple species and verified the accuracy of our results by comparing them to traditional MSA and using biological evidence from the hypervariable regions.

There are many possible applications such as identification of identical DNA fragments and their positions within multiple sequences for DNA barcoding studies. Our methods can reduce the search space from the entire length of DNA sequences to just those regions that are part of stronger quasi-alignments. Other applications might include identification of sequences from their quasi-alignment models and finding interesting regions within sequences, such as those with high GC content.

In this paper we have restricted our discussion to creating non-overlapping segments. For dealing with sequences which contain a larger amount of insertions and deletions or for classification of shorter fragments sampled randomly from the sequence, it is necessary to use overlapping segments while constructing GenModels. The runtime complexity increases only by the constant factor *l*, the segment length. We are currently working on expanding the **QuasiAlign **package to support use of overlapping segments.

## Competing interests

The authors declare that they have no competing interests.

## Authors' contributions

Both authors contributed equally to this paper. All authors read and approved the final manuscript.

## Declarations

The publication costs for this article were funded by the corresponding authors.

This article has been published as part of BMC Bioinformatics Volume 14 Supplement 11, 2013: Selected articles from The Second Workshop on Data Mining of Next-Generation Sequencing in conjunction with the 2012 IEEE International Conference on Bioinformatics and Biomedicine. The full contents of the supplement are available online at http://www.biomedcentral.com/bmcbioinformatics/supplements/14/S11.
